# The Phenotypic Spectrum of Patients with PHARC Syndrome Due to Variants in *ABHD12*: An Ophthalmic Perspective

**DOI:** 10.3390/genes12091404

**Published:** 2021-09-11

**Authors:** Xuan-Thanh-An Nguyen, Hind Almushattat, Ine Strubbe, Michalis Georgiou, Catherina H. Z. Li, Mary J. van Schooneveld, Inge Joniau, Elfride De Baere, Ralph J. Florijn, Arthur A. Bergen, Carel B. Hoyng, Michel Michaelides, Bart P. Leroy, Camiel J. F. Boon

**Affiliations:** 1 Department of Ophthalmology, Leiden University Medical Center, 2333 ZA Leiden, The Netherlands; x.nguyen@lumc.nl; 2 Department of Ophthalmology, Amsterdam UMC, Academic Medical Center, 1105 AZ Amsterdam, The Netherlands; h.almushattat@amsterdamumc.nl (H.A.); m.j.vanschooneveld@amsterdamumc.nl (M.J.v.S.); 3 Department of Ophthalmology, Ghent University and Ghent University Hospital, Corneel Heymanslaan 10, 9000 Ghent, Belgium; Ine.Strubbe@uzgent.be (I.S.); inge.joniau@uzgent.be (I.J.); bart.leroy@ugent.be (B.P.L.); 4 UCL Institute of Ophthalmology, University College London, London EC1V 9EL, UK; michalis.georgiou.16@ucl.ac.uk (M.G.); michel.michaelides@ucl.ac.uk (M.M.); 5 Moorfields Eye Hospital NHS Foundation Trust, 162 City Road, London EC1V 2PD, UK; 6 Department of Ophthalmology, Radboud University Medical Center, 6525 GA Nijmegen, The Netherlands; Catherina.Li@radboudumc.nl (C.H.Z.L.); Carel.Hoyng@radboudumc.nl (C.B.H.); 7 Donders Institute for Brain, Cognition and Behaviour, 6525 HR Nijmegen, The Netherlands; 8 Center for Medical Genetics, Ghent University and Ghent University Hospital, 9000 Ghent, Belgium; elfride.debaere@ugent.be; 9 Department of Clinical Genetics, Amsterdam UMC, Academic Medical Center, 1105 AZ Amsterdam, The Netherlands; r.j.florijn@amsterdamumc.nl (R.J.F.); aabergen@amsterdamumc.nl (A.A.B.); 10 Netherlands Institute for Neuroscience, Institute of the Royal Netherlands Academy of Arts and Sciences (KNAW), 1105 BA Amsterdam, The Netherlands; 11 Division of Ophthalmology, The Children’s Hospital of Philadelphia, Philadelphia, PA 19104, USA; 12 Center for Cellular & Molecular Therapeutics, The Children’s Hospital of Philadelphia, Philadelphia, PA 19104, USA

**Keywords:** PHARC syndrome, *ABHD12*, polyneuropathy, hearing loss, ataxia, retinitis pigmentosa, cataract

## Abstract

This study investigated the phenotypic spectrum of PHARC (polyneuropathy, hearing loss, ataxia, retinitis pigmentosa and early-onset cataract) syndrome caused by biallelic variants in the *ABHD12* gene. A total of 15 patients from 12 different families were included, with a mean age of 36.7 years (standard deviation [SD] ± 11.0; range from 17.5 to 53.9) at the most recent examination. The presence and onset of neurological, audiological and ophthalmic symptoms were variable, with no evident order of symptom appearance. The mean best-corrected visual acuity was 1.1 logMAR (SD ± 0.9; range from 0.1 to 2.8; equivalent to 20/250 Snellen) and showed a trend of progressive decline. Different types of cataract were observed in 13 out of 15 patients (87%), which also included congenital forms of cataract. Fundus examination revealed macular involvement in all patients, ranging from alterations of the retinal pigment epithelium to macular atrophy. Intraretinal spicular hyperpigmentation was observed in 7 out of 15 patients (47%). From an ophthalmic perspective, clinical manifestations in patients with PHARC demonstrate variability with regard to their onset and severity. Given the variable nature of PHARC, an early multidisciplinary assessment is recommended to assess disease severity.

## 1. Introduction

PHARC (polyneuropathy, hearing loss, ataxia, retinitis pigmentosa (RP) and early-onset cataract) is an acronym for a rare, neurodegenerative disease caused by biallelic variants in the *ABHD12* gene [1,2]. *ABHD12* is located on chromosome 20 and encodes the α/β-hydrolase domain-containing protein 12 (ABHD12), which is highly expressed in the central nervous system (CNS) and plays a vital role in lipid metabolism. In vitro, ABHD12 inactivates the main endocannabinoid lipid transmitter 2-arachidonyl glycerol (2-AG), which acts on the cannabinoid receptors 1 and 2 (CB1 and CB2) by converting the 2-AG into the metabolites arachidonate and glycerol [3,4]. In vivo, ABHD12 serves as a lyso-phosphatidylserine (lyso-PS) lipase, which degrades lyso-PS that is biosynthesized by *ABHD16A* [5]. Disruption of ABHD12 in mice leads to (i) accumulation of lyso-PS in the cerebellum breaching the homeostatic threshold, inducing continuous stimulation of the Purkinje neurons, leading to deregulated cerebellar activity and (ii) increased levels of microglial activation and inflammation [5,6,7]. Accompanying this inflammatory response in mice are behavioral deficits, including sensorimotor defects and hearing loss, which resembles the phenotype described in patients with PHARC syndrome [5,6,7].

Patients with PHARC syndrome demonstrate clinical variability with regard to disease onset, severity and progression [1,8,9,10]. Polyneuropathy is typically one of the first findings in patients with PHARC syndrome, which usually manifests in childhood. Early signs of polyneuropathy include distal muscle weakness, sensory disturbances, pes cavus and Achilles tendon contractures [1,9,11]. Sensorineural hearing loss is present in most patients with PHARC, with severity varying from moderate hearing loss to profound deafness [1,8]. RP is reported in the second or third decade of life, with fundoscopy showing optic disc pallor, retinal vessel attenuation and intraretinal specular hyperpigmentation [1,8]. As a result of RP, patients experience night blindness, constricted visual fields and, ultimately, central vision loss when retinal degeneration reaches the fovea [12]. While PHARC syndrome encompasses neurological, auditory and ophthalmic findings, not all of these findings are necessarily present at initial presentation [1,2]. Depending on the presenting symptoms, patients may first be misdiagnosed with other neurodegenerative diseases that give rise to roughly similar phenotypes, such as Charcot–Marie–Tooth, Usher type 3 and adult Refsum disease [1,11].

Because of the considerable challenges of diagnosing PHARC syndrome in patients, it is pivotal to gain more insight into the clinical and genetic characteristics of this neurodegenerative disease. From an ophthalmic perspective, little is known about the retinal phenotype in PHARC syndrome. Expanding our clinical and genetic knowledge on PHARC syndrome may provide insights into its onset, its natural history and the existence of genotype–phenotype correlations. In turn, this would ameliorate our understanding of the function of ABHD12 in humans, which may aid in opening avenues for potential future treatment strategies in the future. To this end, we describe the ophthalmic and associated clinical findings in patients with PHARC syndrome with biallelic *ABHD12* variants.

## 2. Materials and Methods

### 2.1. Patient Population

Clinical data were retrospectively obtained from the Amsterdam University Medical Centers (The Netherlands), Radboud University Medical Center (The Netherlands), Ghent University Hospital (Belgium) and Moorfields Eye Hospital (United Kingdom). Patients with biallelic (likely) pathogenic variants in *ABHD12* were included in the study. A clinical diagnosis of PHARC was based on the presence of variable combinations of polyneuropathy, HL, ataxia, RP and cataract. Five patients (D-6, F-8, G-9, H-10 and I-11) have been described previously [10,13]. This study was approved by the Medical Ethics Committee of the Erasmus University Medical Center (MEC-2010-359; approval date, 10 October 2013) and by the local review board of the Amsterdam University Medical Centers (approval date, 18 November 2013). The study adhered to the Tenets of the Declaration of Helsinki and most patients provided informed consent for the use of their clinical data for research purposes. For Belgian patients, the local ethics committee waivered the need for informed consent on the condition of pseudonymization.

### 2.2. Data Collection

Data were obtained through standardized review of medical records and included sociodemographic information, medical history, age at onset, (previous) clinical diagnosis, best-corrected visual acuity (BCVA), slit-lamp examination, fundus findings, full-field electroretinogram (ffERG), macular region on spectral-domain optical coherence tomography (SD-OCT) imaging and fundus autofluorescence (FAF) imaging. Neurological and audiological data, if available, comprised of a complete physical examination, nerve conduction studies, magnetic resonance brain imaging (MRI) and audiometric testing.

### 2.3. Genetic Analysis

Genomic DNA was obtained from peripheral blood samples using standard protocols. Genetic analysis was performed at each respective center and was performed using a combination of Sanger sequencing and next-generation sequencing, which included targeted gene panel testing, whole exome sequencing and whole genome sequencing. Nucleotide numbering was based on the coding reference NM_001042472.3. For missense variants, pathogenicity predictions from SIFT, Align GVGD and Polyphen-2 were compared (Supplementary Table S1). The pathogenicity of each variant was assessed and classified according to the American College of Medical Genetics and Genomics (ACMG) guidelines [14].

### 2.4. Statistical Analysis

Data analysis was performed using R version 3.6.2 (R Foundation for Statistical Computing, Vienna, Austria). The normality of data was analyzed using the Shapiro–Wilk test and was also visually plotted. Continuous data were either presented as mean, standard deviations (SD) and range, whereas categorical data were presented as frequencies and percentages. BCVA data was converted to logarithm of the minimum angle of resolution (logMAR) values for statistical analysis. For vision categories of counting fingers, hand movements, light perception and no light perception, logMAR values of 2.6, 2.7, 2.8 and 2.9 were used, respectively [15].

## 3. Results

### 3.1. Patient Characteristics

A total of 15 patients from 12 different families were included in this study. An overview of the clinical and genetic characteristics of included patients is provided in Table 1. Most patients were male (*n* = 12; 80%) and the mean age at the most recent examination was 36.7 years (SD ± 11.0; range from 17.5 to 53.9). Previous (mis)diagnoses, available for 13 patients (87%), included forms of retinal degeneration (e.g., non-syndromic RP or Usher, *n* = 9; 69%), Charcot–Marie–Tooth (*n* = 2; 17%), spinocerebellar ataxia (*n* = 1; 8%) and optic neuropathy (*n* = 1; 8%). Phytanic acid levels were also assessed in 5 patients (A-1, B-2, C-3, C-4 and C-5) to rule out adult Refsum disease.

In total, 13 different *ABHD12* variants were found in this cohort, 3 of which were missense variants, 3 splice site variants, 4 nonsense variants and 3 frameshift variants (Table 1 and Supplementary Table S1). The most common variant in this cohort was the frameshift variant c.337_338delGAinsTTT, which was present in more than half of the cohort in either homozygous or compound heterozygous form. This variant in exon 3 was predicted to result in a substitution of asparagine with phenylalanine at codon 113, introducing a premature termination codon (p.[Asp113Phefs*15]).

### 3.2. Clinical Examination

The onset of neurological, auditory and ophthalmic symptoms was variable, with no apparent order of symptom occurrence (Table 1). Results from nerve conduction studies were available for nine patients (60%), which revealed various degrees of demyelinating polyneuropathy, even in an asymptomatic patient. Patient C-3 had no subjective complaints of sensory or motor deficits, despite both of his siblings (patients C-4 and C-5) being diagnosed with severe demyelinating polyneuropathy in childhood years. Still, upon neurological evaluation, a subtle foot drop and absent Achilles tendon reflexes were detected, with nerve conduction studies revealing a demyelinating polyneuropathy. Hearing loss was not subjectively present in two patients (patients C-3 and H-10), although formal audiometric testing results were not available in these patients. Similarly, the presence of ataxia was observed in less than half of the cohort, although the absence of ataxia could not be excluded in five patients as neurological examination was not performed or data were not available. MRI was performed in six patients (40%; patients A-1, C-3, C-4, C-5, E-7, J-12 and J-13), with signs of cerebellar atrophy in one patient with ataxia (patient C-5) and two patients without ataxia (J-12 and J-13).

The ophthalmic findings in this cohort at the last visit are described in Table 2. Loss of BCVA was observed in all patients (100%), with a mean BCVA of 1.1 logMAR (SD ± 0.9; range from 0.1 to 2.8), which is equivalent to approximately 20/250 Snellen acuity. Four patients (A-1, B-2, E-7 and J-13), who carried the variant c.337_338delGAinsTTT in either homozygous or compound heterozygous form, had relatively preserved BCVA (BCVA ≥ 20/40 Snellen in the better-seeing eye). In contrast, the remaining patients, despite being in a similar age range, had visual acuities that could be classified as low vision (BCVA < 20/70 Snellen in the better-seeing eye) or worse. Patients with preserved BCVA were not significantly younger than those with low vision (−4.6 years, *p* = 0.496; independent *t*-test).

**Figure 1:**
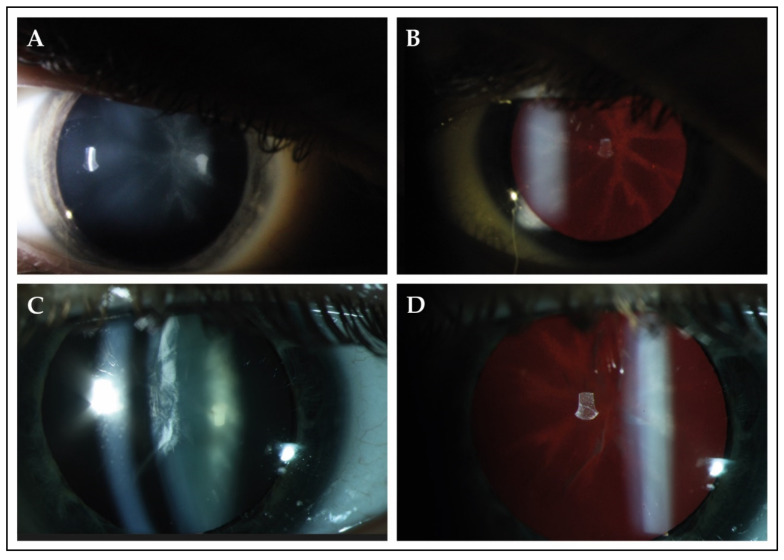
Slit-lamp findings in 2 patients with PHARC syndrome. (**A**,**B**) Slit-lamp photographs of the right eye of patient J-13 at the age of 17. Best-corrected visual acuity was 20/50 Snellen in this eye. Direct illumination demonstrated the presence of cataract in the posterior surface of the lens. Retroillumination revealed that the observed opacity followed a star-shaped distribution, which seemed to delineate the crystalline lens sutures of the posterior cortex. (**C**,**D)** The right eye of patient L-15 (age 37) showed opacities in both the anterior and posterior cortex. Best-corrected visual acuity was 20/100 during this visit. Retroillumination showed anterior cortical cataract and a star-shaped opacity in the posterior surface.

Slit-lamp examination revealed cataracts in 13 patients (87%), of whom 10 patients (77%) underwent uncomplicated cataract extraction. Various types of cataract were observed in this cohort, which also included congenital forms of cataract (Table 1). In four patients (patients C-5, J-12, J-13 and L-15), lens opacities were located in the posterior surface of the lens and followed a star-shaped distribution (Figure 1). Patients underwent their first cataract extraction and intraocular lens implementation at a mean age of 30.3 (SD ± 8.8; range from 19.0 to 44.0).

**Figure 2:**
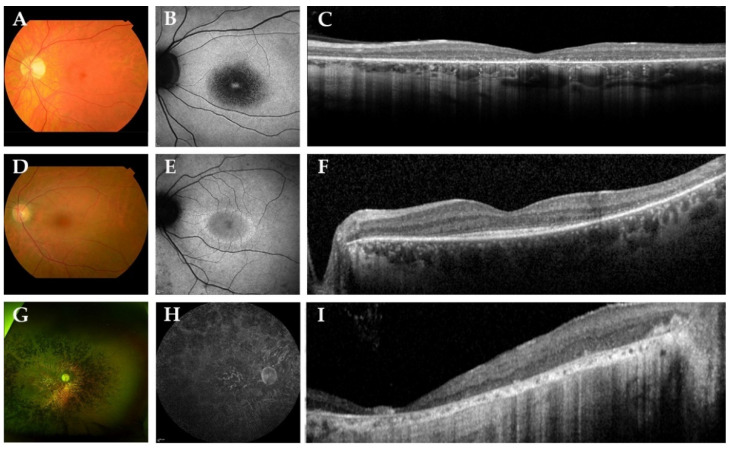
Representative color fundus photographs with corresponding fundus autofluorescence (FAF) and spectral-domain optical coherence tomography (SD-OCT) images in this cohort of patients with biallelic *ABHD12* variants. (**A**–**C**) The left eye of patient D-6, a 42-year-old man with Snellen best-corrected visual acuity (BCVA) of 20/400. Fundus photography revealed a slightly pale optic disc, atrophic macular changes and retinal pigment epithelium (RPE) changes in the midperipheral retina, in the absence of spicular hyperpigmentation. FAF imaging showed a region of hypo-autofluorescence (hypo-AF) in the central macula with a hyper-autofluorescent (hyper-AF) spot in the fovea. On SD-OCT, loss of the external limiting membrane and ellipsoid zone was observed. (**D**–**F**) The left eye of a 36-year-old woman, patient E-7, with Snellen BCVA of 20/29. Fundus imaging showed macular and midperipheral alterations, with no evident spicular hyperpigmentation. On FAF imaging, hypo-AF zones of RPE degeneration were present outside the macula and a macular hyper-AF ring was observed. SD-OCT showed preservation of the outer retinal layers in the (para)fovea. (**G**–**I**) Patient F-8, a 53-year-old man with light perception visual acuity, showed extensive degeneration across the entire retina with dense spicular hyperpigmentation reaching the posterior pole. FAF imaging demonstrated generalized hypo-AF due to the extensive RPE atrophy. SD-OCT showed marked chorioretinal atrophy.

In Figure 2, we present representative fundus and multimodal imaging findings of this cohort. Fundus examination revealed signs of retinal degeneration in all patients, although a clinical hallmark of RP—intraretinal spicular hyperpigmentation—was only observed in 7 out of 15 patients (47%; Table 2). Patients with intraretinal spicular hyperpigmentation had worse logMAR BCVA than those without pigmentation (+0.9 logMAR BCVA, *p* = 0.019; independent *t*-test). Macular involvement was present in all patients (100%), ranging from retinal pigment epithelium alterations to macular atrophy. Full-field electroretinography data were available for 10 patients (67%), showing a rod-cone dystrophy pattern (*n* = 8; 80%) or minimal scotopic and photopic responses (*n* = 2; 20%) (Supplemental Figure S1).

FAF imaging was available for 13 out of 15 patients (87%). A common finding was the presence of a hypo-autofluorescent area in the central macula in 6 out 13 patients (46%; Figure 2B). Other FAF patterns included a macular hyperautofluorescent ring in four patients with relatively preserved BCVA (patients A-1, B-2, E-7 and J-13; Figure 2E) and generalized, mottled hypo-autofluorescence in patient F-8, who had light perception vision (Figure 2H). A description of the other FAF patterns observed is provided in Table 2. SD-OCT imaging showed degeneration of the outer retinal layers, the external limiting membrane and the ellipsoid zone, in all patients outside the central macula (100%) and, to various extents, within the central macula. The mean central retinal thickness in this cohort was 109 μm (SD ± 63.4; range from 25.0 to 207.5). Preservation of the outer retinal layers in the central macula was observed in the four patients with preserved BCVA (patients A-1, B-2, E-7 and J-13; Figure 2F). Cystoid macular edema was observed in three patients (20%), which resolved several years later without treatment.

## 4. Discussion

In this retrospective study, we report the genetic and clinical characteristics of 15 patients with PHARC syndrome with variable severity, caused by variants in the *ABHD12* gene. This study aimed to expand our clinical knowledge on PHARC syndrome, as it is a rare neurodegenerative disease with less than 50 cases currently described in the literature [11,16]. The advent of next-generation sequencing has made it possible to identify disease-causing variants on novel genes at a rapid pace and the ongoing improvements in this technology may lead to an increased detection of patients with biallelic *ABHD12* variants/PHARC syndrome in the future [17]. In our study, we found 13 different variants in *ABHD12*, with the most common variant being c.337_338delGAinsTTT. This is a relatively well-known variant, as it was the first described variant in the Norwegian cohort of Fiskerstrand et al., which may suggest a common European ancestry [1,2]. No evident genotype–phenotype correlation could be established, although four patients with this variant on one or two allele(s) had relatively preserved BCVA and intact ELM/EZ, compared with the other patients in this cohort, which suggests a relatively milder ocular phenotype. However, we were unable to correlate ophthalmic findings with neurological or audiological assessments (e.g., nerve conduction studies, MRI and audiograms), as these evaluations were either (i) not (routinely) performed, (ii) performed in a much earlier or later stage of disease than ophthalmic examinations, (iii) or not available due to the retrospective nature of this study. In order to establish potential genotype–phenotype correlations, quantitative data from all involved clinical disciplines need to be obtained within similar time frames, which requires a coordinated interdisciplinary approach. This remains challenging, as patients show variability in the manifestation of neurological, audiological and ophthalmic symptoms, which may result in misdiagnosis, delayed diagnosis and delayed referral for assessment of these symptoms. This was also the case for our cohort, as the presence and onset of neurological, audiological and ophthalmic symptoms were variable, even in those from within the same family. No evident order of appearance of symptoms was observed, as neurological symptoms could precede ophthalmic symptoms and vice versa. This variable onset of the different symptoms resulted in different diagnoses at the initial visit, including (non-)syndromic forms of RP, Charcot–Marie–Tooth and adult Refsum disease, consequently delaying an accurate diagnosis of PHARC syndrome. A patient’s self-reported onset of symptoms may not be a particularly reliable indicator for the presence of disease, as self-reported data are susceptible to recall bias and patients may be asymptomatic in early disease stages [18]. The latter is illustrated by patient C-3, who was asymptomatic for neurological deficits, but was revealed to have demyelinating sensorimotor polyneuropathy on nerve conduction studies. Similarly, the 47-year-old patient A-1 reported no subjective symptoms of RP, although a rod-cone degeneration pattern of the retina was established during full-field ERG. Most likely, disease progression must reach a certain threshold before noticeable symptoms are reported by patients. Establishing the onset and severity of *ABHD12*-associated clinical findings objectively is crucial to determine the natural history of PHARC syndrome, as well as to establish genotype–phenotype correlations. Ideally, patients should undergo objective testing from all involving clinical disciplines at the time of diagnosis and they should also be monitored over a prolonged period of time.

In all patients with available neurological data, demyelinating polyneuropathy was detected in all, with variable presence of ataxia and cerebellar atrophy. These findings are consistent with previous studies on PHARC syndrome [1,11]. The exact etiology of neurologic deficits in patients with PHARC remains unclear, but previous studies have shown that accumulation of lyso-PS in the cerebellum due to disruptive ABHD12 leads to increased levels of microglial activation and neuroinflammation, which is the presumed cause for neurological deficits in *abhd12* knockout mice [5,6,7]. Ataxia without cerebellar atrophy might be explained based on the cellular localization of ABHD12 in the Purkinje neurons. When ABHD12 activity is absent, the Purkinje neurons are constantly stimulated by the accumulated lyso-PS, resulting in deregulated cerebellar function [19]. Consistent with previous studies, we found that hearing loss was present in the majority of patients in childhood/early adolescence [1,10,20,21]. To date, it remains unclear where ABHD12 localizes and interacts in the inner ear, which is crucial for determining the cause of hearing loss in patients with PHARC syndrome. Previous studies have shown that microglial cells are in abundance in the inner ear and can be modulated by distant inflammation by the central nervous system [22]. It could be that microglial cells in the inner ear undergo similar inflammation processes as in the central nervous system due to disruptive ABHD12, with consequent hearing loss, although this hypothesis requires further testing.

From an ophthalmic perspective, we found that the majority of patients (57%) did not exhibit typical intraretinal spicular hyperpigmentation as a characteristic sign of RP on fundus examination, despite patients experiencing subjective symptoms of RP. This phenotype resembles an atypical variant of RP without pigmentation [23]. These patients may demonstrate an early remodeling stage of RP, in which pigmented RPE cells still need to migrate to the inner retina, before their demise leads to the classic sign of spicular hyperpigmentation as seen on fundoscopy [23,24,25]. Longer follow-up in patients with PHARC syndrome is required to confirm this hypothesis. Despite the absence of spicular pigment migration in patients, the diagnosis rod-cone dystrophy could still be established using ffERG, which highlights the importance of electrophysiological testing in this disease. FAF patterns were variable, although a common finding was the early presence of a hypo-AF region in the macula, indicative of early macular involvement and early loss of visual acuity. The extent of macular damage was not assessed in this study, but could potentially be measured using multifocal/pattern ERG or microperimetry, the latter being commonly used as an outcome measure in gene therapy trials [26,27]. Consistent with FAF findings, SD-OCT showed degeneration of the outer retinal layers in all patients, with preservation of the ELM/EZ at (para)fovea in only four patients who had relatively preserved BCVA values despite being in similar age ranges as those with no preservation. Therefore, multimodal imaging techniques—in conjunction with more sensitive outcome measures of the macula—are valuable tools to assess the disease severity in the retina and can likely be used to monitor disease progression in patients with PHARC syndrome.

Visually significant cataract was observed between the second and fourth decade of life in the majority of patients, which is within the reported age range of cataract found in patients with non-syndromic forms of RP [28,29,30]. The exact pathogenesis of cataract formation in patients with RP is still unknown, although it has been hypothesized that the degenerative retina of patients with RP increases the levels of proinflammatory cytokines and chemokines in the vitreous, which may reach and change the homeostatic state of the natural lens, resulting in formation of cataract [30,31]. It is unclear whether this hypothesis on cataract morphogenesis is also applicable to patients with *ABHD12* variants, as several patients in this cohort were diagnosed with cataract types that suggest a congenital origin [32]. A single case of posterior polar cataract was also reported in the study of Fiskerstrand and colleagues, although a description of cataract morphology was not provided for the other patients of this cohort [1]. In addition, we reported a star-shaped cataract in the posterior cortex of the lens in several related and unrelated patients, that presumably delineated the crystalline lens sutures of the posterior cortex, which is also suggestive for a congenital origin [32,33].

The molecular and cellular basis for degeneration in the retina and cataract formation due to *ABHD12* variants remains to be elucidated. To our knowledge, while the expression of *ABHD12* in the brain has been established, limited studies have investigated the expression of *ABHD12* in the neurosensory retina or in the natural lens of the eye [1]. Previous studies in *abhd12* knockout mice showed no degenerative changes in the retina and no lens opacities, despite exhibiting neurological and auditory deficits, which suggests limited expression of *ABHD12* in the eye [19]. Naturally, these findings should be interpreted with caution, as recapitulating human diseases with murine models remains challenging. By analyzing the Human Protein Atlas, we observed some degree of *ABHD12* expression in photoreceptor, bipolar and horizontal cells (available from http://www.proteinatlas.org, accessed on 2 July 2021) [34]. A high expression of *ABHD12* is found in microglial cells, which are the resident immune cells of the brain and are also present in the plexiform layers of the retina [1,35]. Microglial cells possibly play an initiating role in the degeneration of the retina in patients with *ABHD12* variants, which aligns with the hypotheses of the microglial cell possibly being one of the driving forces behind neurodegeneration in the central nervous system and inner ear [1,8]. Establishing the causative agent for neurodegeneration in patients with *ABHD12* variants is pivotal for the development of treatment modalities for this neurodegenerative disease. For inherited retinal dystrophies, promising results have been achieved with gene therapy, resulting in improvements in visual function [36,37]. Given the size of the primary transcript of *ABHD12* (1.1 kb, NM_001042472.3), *ABHD12* likely fits into AAV vectors and is therefore a potential candidate for gene augmentation therapy. If the underlying cause of PHARC syndrome has a metabolic and/or immunological basis, suppressing or inhibiting targets in relevant pathways, such as the previously mentioned lyso-PS pathway, could prove to be an attractive alternative approach [19]. Further research into the molecular and biochemical basis of *ABHD12* would likely determine the most optimal path for treatment.

In conclusion, we report the phenotype of patients with PHARC syndrome due to biallelic *ABHD12* variants. Rod-cone dystrophy is present in all patients with PHARC syndrome with early macular involvement, although this finding may vary widely in its onset and severity. Given the variability in symptoms and clinical findings in patients with PHARC syndrome, patients should be evaluated in a multidisciplinary setting, involving ophthalmologists, neurologists, audiologists/otologists and geneticists, when PHARC syndrome is either suspected or genetically confirmed.

## Figures and Tables

**Table 1:** Genetic and clinical characteristics at last examination of patients with biallelic *ABHD12* variants.

Family-ID	Sex, Age	Genetic Analysis		Presence of PHARC Syndrome Symptoms and Age at Symptom Onset/Diagnosis (Years)				
		Allele 1/Allele 2	Protein Change	Polyneuropathy	Hearing Loss	Ataxia	Retinitis Pigmentosa	Cataract
A-1	M, 47	c.337_338delGAinsTTT/ c.1075del	p. (Asp113Phefs*15)/p. (Val359Phefs*27)	Pes cavus, hammertoes, distal sensory loss and absent tendon reflexes; age 8	Yes; age 28	Yes; age 8	Asymptomatic, detected during electrophysiological testing at age 45	Yes; age 36
B-2	F, 32	c.337_338delGAinsTTT/ c.337_338delGAlinsTTT	p. (Asp113Phefs*15)/p. (Asp113Phefs*15)	Yes; childhood	Yes; age 17	Yes; age 45	Reduced visual acuity; age 32	Posterior subcapsular cataract; age 32
C-3 *	M, 33	c.337_338delGAinsTTT/c.423-1_425del	p. (Asp113Phefs*15)/p. (?)	Asymptomatic; but detected during examination at age 27	No ¶	Yes; age 27	Night blindness; age 14	Sutural cataract; age 3
C-4 *	M, 33	c.337_338delGAinsTTT/c.423-1_425del	p. (Asp113Phefs*15)/p. (?)	Distal muscle weakness and sensory loss; childhood	Yes; NA	Yes; age 27	Night blindness; age 21	Sutural cataract; age 3
C-5 *	M, 38	c.337_338delGAinsTTT/c.423-1_425del	p. (Asp113Phefs*15)/p. (?)	Abnormal gait pattern; childhood	Yes, 20	Yes; age 31	Night blindness	Star-shaped cataract; age 4
D-6	M, 42	c.477G > A/c.557G > C	p. (Trp159*)/p. (Arg186Pro)	Distal sensory loss and reduced tendon reflexes; age 35	Yes; age 36	Yes; NA	Reduced visual acuity; age 29	Cortical cataract; age 29
E-7 ^†^	F, 36	c.337_338delGAinsTTT/ c.337_338delGAlinsTTT	p. (Asp113Phefs*15)/p. (Asp113Phefs*15)	NA ^‡^	Yes; age 12	Yes; NA	Visual field loss; age 31	Posterior subcapsular cataract; age 32
F-8	M, 53	c.784C > T/c.867 + 5G > A	p. (Arg262*)/ p. (?)	Distal sensory loss; age 53 ^‡^	Yes; age 20	NA	Reduced visual acuity; age 18	No
G-9	M, 34	c.620-2A > G/c.620-2A > G	p. (?)/p. (?)	Lower limb muscle weakness; age 31 ^‡^	Yes; age 20	NA	Reduced visual acuity and night blindness; age 22	Yes; age 26
H-10 ^†^	M, 22	c.193C > T/c.193 C > T	p. (Arg65*)/p. (Arg65*)	Lack of coordination; age 7 ^‡^	No ¶	NA	Reduced visual acuity; age 16	No
I-11 ^†^	M, 53	c.374C > T/c.1154T > C	p. (Thr125Met)/p. (Leu385Pro)	NA, but epilepsy and learning difficulties ^‡^	Yes; age 44	NA	Reduced visual acuity and night blindness; age 30	Posterior polar cataract; age 41
J-12 *	M, 20	c.337_338delGAinsTTT/c.337_338delGAinsTTT	p. (Asp113Phefs*15)/p. (Asp113Phefs*15)	Yes; age 20	Yes; age 16	No	Night blindness; age 16	Star-shaped cataract; age 17
J-13 *	M, 17	c.337_338delGAinsTTT/c.337_338delGAinsTTT	p. (Asp113Phefs*15)/p. (Asp113Phefs*15)	Yes; age 18	Yes; age 10	No	Reduced visual acuity; age 10	Star-shaped cataract; age 10
K-14	F, 46	c.1063C > T/c.1063C > T	p. (Arg355*)/p. (Arg355*)	Yes; age 47	Yes; NA	Yes; NA	Yes; NA	Cerulean cataract, NA
L-15	M, 39	c.337_338delGAinsTTT/c.341dup	p. (Asp113Phefs*15)/p. (Leu114Phefs*14)	NA ^‡^	Yes; age 33	NA	Night blindness; age 23	Star-shaped cataract; age 29

Nucleotide numbering is based on reference sequence NM_001042472.3. * Patients C-3, C-4 and C-5 are siblings and patients J-12 and J-13 are siblings. ^†^ Consanguineous parents. ^‡^ Neurological evaluation/electrophysiological testing was not performed or available in these patients. ¶ Patients reported no subjective hearing loss, although audiometric testing results were not available. F = female; M = male; NA = data not available.

**Table 2:** Summary of ophthalmic findings at the most recent examination in this cohort of patients with biallelic *ABHD12* variants.

Family-ID	Sex, Age	BCVA (OD; OS)	Lens Status; Age at First Surgery	ffERG	Fundus Findings			
					Macular Changes	Bone Spicules	Spectral-Domain Optical Coherence Tomography	Fundus Autofluorescence
A-1	M, 47	20/22; 20/22	Pseudophakic; surgery at age 36	RCD	RPE alterations	No	Epiretinal membrane, degeneration of the outer retina with preservation of ELM and EZ at the (para)fovea	Hypo-AF regions in midperiphery with a macular hyper-AF ring
B-2	F, 32	20/25; 20/25	Pseudophakic; surgery at age 32	RCD	RPE alterations	No	Degeneration of the outer retina with preservation of ELM and EZ at the (para)fovea	Central hypo-AF surrounded by a hyper-AF ring
C-3	M, 33	20/200; 20/200	Pseudophakic; surgery at age 26	NA	Atrophy	Yes	Degeneration of the outer retina	NA
C-4	M, 33	20/125; 20/100	Pseudophakic; surgery at age 21	MR	Atrophy	Yes	Epiretinal membrane, degeneration of the outer retina, CME ODS at age 29, resolved at age 31	Hypo-AF lesions in the midperiphery with hyper-AF changes in the central macula
C-5	M, 38	20/134; 20/134	Pseudophakic; surgery at age 19	MR	Atrophy	Yes	Epiretinal membrane, degeneration of the outer retina, CME ODS at age 30, resolved at age 32	NA
D-6	M, 42	20/400; 20/400	Cortical cataract	RCD	Atrophy	No	Degeneration of the outer retina	Central hypo-AF with a hyper-AF foveal spot
E-7	F, 36	20/29; 20/29	Pseudophakic; surgery at age 29	RCD	RPE alterations	No	Degeneration of the outer retina with preservation of ELM and EZ at the (para)fovea	Hypo-AF regions in midperiphery with a macular hyper-AF ring
F-8	M, 53	LP; LP	Clear lens	NA	Atrophy	Yes	Extensive atrophy of all retinal layers	Generalized hypo-AF
G-9	M, 34	20/400; 20/400	Pseudophakic; surgery at age 34	RCD	Atrophy	No	Extensive atrophy of all retinal layers at the fovea, with relatively preserved layers in the perifovea	Central hypo-AF
H-10	M, 22	20/240; 20/240	Clear lens	RCD	Bull’s eye	No	Degeneration of the outer retina	Central hypo-AF
I-11	M, 53	HM; HM	Pseudophakic; surgery at age 44	NA	Atrophy and Macular hole OS	Yes	Degeneration of the outer retina. Macular hole OS.	Mottled patches of hypo-AF in nasal region with hypo-AF in the central macula
J-12	M, 20	20/200; 20/200	Pseudophakic; surgery at age 20	NA	Atrophy	No	Degeneration of the outer retina.	Central hypo-AF with hyper-AF borders
J- 13	M, 17	20/50; 20/40	Star-shaped cataract	NA	Atrophy	No	Degeneration of the outer retina with preservation of ELM and EZ at the (para)fovea	Hyper-AF ring surrounded by a larger hyper-AF ring
K-14	F, 46	HM; 20/400	Cerulean cataract	RCD	Atrophy	Yes	Degeneration of the outer retina.	Central hypo-AF with a hyper-AF foveal spot. Several hypo-AF lesions along the superior vascular arcade.
L-15	M, 39	20/134; 20/200	Pseudophakic; surgery at age 39	RCD	Atrophy	Yes	Degeneration of the outer retina. CME ODS at age 33, resolved at age 39	Generalized hypo-AF with preserved AF in the central macula.

## Supplementary Materials

The following are available online at https://www.mdpi.com/article/10.3390/genes12091404/s1, Table S1: Overview of variants found in *ABDH12*. Figure S1. Full-field electroretinography results of a patient with PHARC syndrome.
