# Supplementary material for: The Phenotypic Spectrum of Patients with PHARC Syndrome Due to Variants in ABHD12: An Ophthalmic Perspective
Source: Genes (Basel). 2021 Sep 11;12(9):1404. doi: 10.3390/genes12091404 (PMC8467809; doi:10.3390/genes12091404)
Supplement: Supplementary file 1 [file genes-12-01404-s001.zip › Supplemental Figure S1. Full-field electroretinography responses of a patient with PHARC syndrome.pdf]

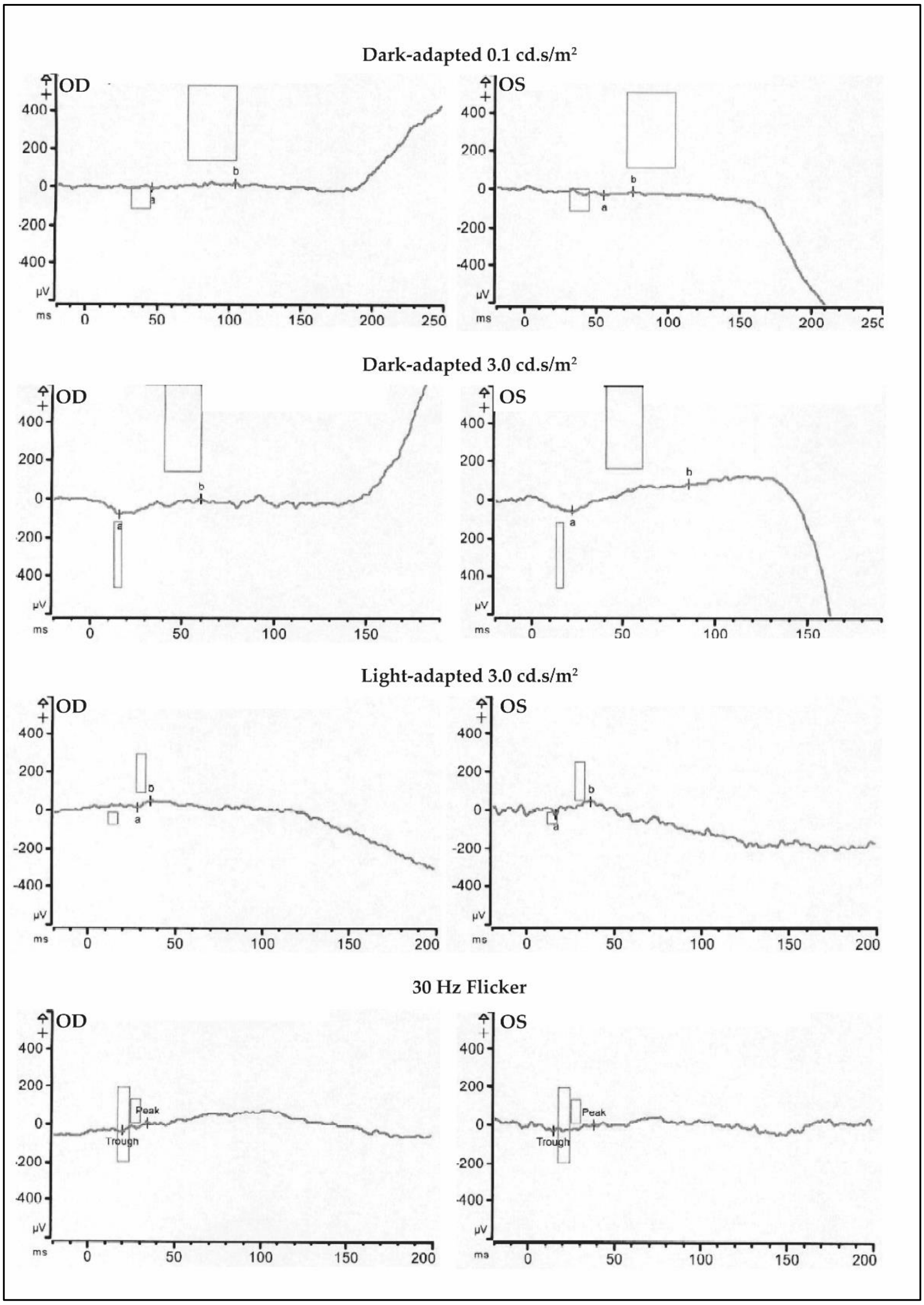

Supplemental Figure S1. Full-field electroretinography (ERG) results of a patient with PHARC (polyneuropathy, hearing loss, ataxia, retinitis pigmentosa and early-onset cataract) syndrome. Full-field ERG responses in both eyes of patient A-1 at the age of 47. Full-field ERGs were performed according to the 'International Society for Clinical Electrophysiology of Vision' standards. Rod responses are (nearly) absent in both eyes, with minimal cone responses measured on light-adapted ERGs. cd.s./m^2 = candela seconds per square meter; Hz = hertz; µV=microvolts; ms = milliseconds; OD = right eye; OS = left eye.
