# Supplementary material for: The Phenotypic Spectrum of Patients with PHARC Syndrome Due to Variants in ABHD12: An Ophthalmic Perspective
Source: Genes (Basel). 2021 Sep 11;12(9):1404. doi: 10.3390/genes12091404 (PMC8467809; doi:10.3390/genes12091404)
Supplement: Supplementary file 1 [file genes-12-01404-s001.zip › Supplemental Table S1. Overview of ABHD12 variants found in this study.pdf]

Supplemental Table S1. *Overview of ABHD12 variants found in this study*

Nucleotide change	Protein effect	Mutation type	Frequency (n =)	SIFT	Align GVGD	Polyphen-2	References
c.193C>T	p.(Arg65*)	Nonsense	2	-	-	-	Eisenberger *et al.* 2012, Igelman *et al.* 2021
c.337_338delinsTTT	p.(Asp113Phefs*15)	Frameshift	13	-	-	-	Fiskerstrand *et al.* 2010
c.341dup	p.(Leu114Phefs*14)	Frameshift	1	-		-	**This study**
c.374C>T	p.(Thr125Met)	Missense	1	Affects protein function	C65	Probably damaging	Igelman *et al.* 2021
c.423-1_425del	p.(?)	Splice site	3	-	-	-	**This study**
c.477G>A	p.(Trp159*)	Nonsense	1	-	-	-	Nishiguchi *et al.* 2014
c.557G>C	p.(Arg186Pro)	Missense	1	Affects protein function	C65	Probably damaging	Nishiguchi *et al.* 2014
c.620-2A>G	p.(?)	Splice site	2	-	-	-	Igelman *et al.* 2021
c.784C>T	p.(Arg262*)	Nonsense	1	-	-	-	Thimm *et al.* 2020, Igelman *et al.* 2021
c.867+5G >A	p.(?)	Splice site	1	-	-	-	Ellingford *et al.* 2019
c.1063C>T	p.(Arg355*)	Nonsense	2	-	-	-	**This study**
c.1075del	p.(Val359Phefs*27)	Frameshift	1	-	-	-	**This study**
c.1154T>C	p.(Leu385Pro)	Missense	1	Affects protein function	C65	Probably damaging	Igelman *et al.* 2021

Nucleotide numbering is based on the reference sequence NM_001042472.3. The Human Gene Mutation Database was accessed on July 5th, 2021.
